# Odontological Society of Pennsylvania

**Published:** 1898-06

**Authors:** 

**Affiliations:** Odontological Society of Pennsylvania


### ODONTOLOGICAL SOCIETY OF PENNSYLVANIA.

The stated meeting of the Odontological Society of Pennsylvania was held December 11, 1897, at 1415 Walnut Street, Philadelphia, President Brubaker in the chair. After the routine business was finished the following remarks and discussion on dental caries took place.

### REMARKS BY DR. C. N. PEIRCE.

Mr. President and Ladies and Gentlemen,—We all recognize that the ideas of the present day have been evolved from the past, and, therefore, I thought it might not be amiss to run hastily over the various facts in the experience of the last century and a half regarding the causes of dental caries.

Those of you who are familiar with the facts remember that Bourdet and Jourdain, in 1754–56, published a paper stating that caries was due to an inflammatory condition of the dentine, and that it was analogous to inflammation of the soft tissues. They believed it arose from want of nutrition.

It is most singular that we have no paper on this subject of any importance from that date until 1835 or 1840. Then Dr. Robertson, of Burlington, announced that dental decay was due to a chemical agent. He stated that he found acids in the mouth, and that when teeth are subject to acids they become devitalized. He published an essay stating that dental decay was due to acid secretions. But he had no knowledge of how the animal matter was disorganized or decomposed.

Following him Dr. George Watt, of Ohio, stated that white decay was due to nitric acid, and that dark decay was due to sulphuric acid, and that where the decay was of different colors it was due to hydrochloric acid; but though he was professor of dentistry in an Ohio college, his assertions made no impression on the profession.

Nothing further was advanced until we had the views of Magitot, who reiterated the views advanced by Robertson, but he made no definite statement as to how dental decay progressed, other than that it was due to acid in the mouth, and gave no explanation of the decompositon of the organic matter of the tooth.

Following Magitot, Desirabode published a short paper in which he stated that decay commenced in the pulp-chamber from the want of nutrition in the dentine, and through a vital action it worked from the pulp-chamber towards the periphery of the tooth. But facts were not consistent with the statement, and it made little or no impression on the profession, the large majority believing that decay was due to chemical action.

Following Desirabode, came John Tomes, the father of our present Charles Tomes, who gave himself to microscopy and dental physiology. He stated that decay was evidently due to a chemical action, and that the acids in the mouth were responsible for it. Still there was no explanation of the decomposition of the organic matter in the tooth.

Following John Tomes, Bridgman, of England, said decay was due to a chemico-electrical action. He took the ground that the tooth was made subject to the influence of acids by having its electrical condition disturbed.

He was sustained by S. B. Palmer, of Syracuse, who holds the ground to-day that the electrical condition of the teeth is responsible for decay. He has written several interesting papers in which he says that it is impossible to estimate how far this condition changes the character of the dentine; and he maintains that it is an abnormal condition that makes the teeth yield to the influence of the acids in the mouth.

This was the condition when Dr. Miller took hold of the subject. Dr. Miller made an examination of the secretions of the mouth, and isolated twenty-two micro-organisms. He found that sixteen of these organisms produced an acid that he believed was instrumental in causing decay. But he was not satisfied that it was the sole cause. He placed teeth in a vessel and inoculated them with these micro-organisms and produced artificial decay, not only of the lime but of the albumen. And so to Dr. Miller we are indebted for our present theory of decay in teeth,—parasitico-chemical,—because produced by micro-organisms and the acid introduced by their multiplication.

This is the theory that we accept to-day, and I believe it is a correct theory, so far as we have any knowledge of caries or decay in the mouth. He said of these twenty-two micro-organisms, sixteen of them would produce either lactic acid or acetic acid, and those two acids, he thinks, are largely instrumental in decalcification.

He named five of the organisms that are found in progressing decay, and from his experiments he has no hesitation in stating that these organisms are instrumental not only in producing the acid and decalcification of the teeth, but also in disorganizing and putrefaction of the organic matter of teeth. And that is the way the case stands at present in regard to decay of the teeth.

Now, a few words in regard to the progress of decay. I think we all accept the statement that decay must commence externally. There must be some opening, so that the secretions of the mouth come in contact with the dentine, before the dentine can become disorganized. Whether enamel is always decalcified, or whether we have organic matter between the enamel fibres disintegrated or loosened, is a fact that we must decide for ourselves in every case. I think every observing dentist will have noticed many cases, especially after typhoid, where an examination under the microscope will show the enamel fibres undecalcified, and the organic matter between these fibres disintegrated.

Now, has this disintegration of the organic material taken place through the action of the acids or through the action of micro-organisms? I do not mean to be understood that all abrasions of the enamel are so produced. We have many cases where the dentine is reached by the decalcification of the enamel fibres. But I also believe that we have a great many where the enamel rods are not decalcified, but the disintegration solely effects the matter between the rods.

I had hoped my friend, Dr. Allan, would be here. He does not believe enamel possesses any vitality. He says, "How can you believe that enamel is not dead the moment it is formed?" His view is endorsed by Dr. Williams, Dr. Andrews, and Dr. Black that formed enamel is dead enamel. I believe that formed enamel is not necessarily dead. I have had evidence that the enamel of a tooth may change in structure and also in its color. And if vital changes take place in any tissue, certainly that tissue is not dead. Chemical changes may take place, but vital changes do not take place in dead matter. I therefore agree with Dr. Truman that formed enamel is not necessarily dead.

(Dr. Peirce then exhibited and explained lantern slides by Gysi.)

Before I close I should like to report the result of a little experiment that may be of some importance. It has long been a question whether a cotton filling would arrest the progress of decay. Now, I placed in these two test-tubes a solution of grape-sugar, and then a plug of cotton in each down to within two inches of the bottom, and inoculated the upper part of the solution with the fungus. You will see that the upper part has fermented, while the lower part is clear. You know some claim that the cotton used for filling should not be absorbent cotton. In one of these vessels there is ordinary cotton, and in the other absorbent cotton, and in each case the cotton equally acts as a bar to bacteria. On one or two occasions patients go home with only cotton in the cavity, and decay has been completely arrested. Of course, the cavity was previously sterilized.

*A Member.*—Without sandarac?

*Dr. Peirce.*—No, the cavity should be first sterilized, then the cotton with sandarac should be placed in.

*Dr. Darby.*—I thought perhaps something was going to be said about some of the systemic conditions and the environments which might possibly change teeth for the better or for the worse.

Dr. Black says that all teeth are equally soft; that the dentine of all teeth is the same in density; that they have the same specific gravity; that a good tooth is no better than a bad tooth; that any filling-material that will save one tooth will save any other; and that there is no choice except on the part of the dentist between lead or tin or gold or amalgam. Well, now, I do not believe that bad teeth are always bad. I do not believe that teeth that are bad in the beginning are bad all through life. I am honest in being mistaken, if I am mistaken, but I believe I have seen changes in the teeth of some of my patients. I call to mind a patient who for ten years suffered from caries of the teeth. He was then leading a sedentary life. He changed his occupation, becoming a railroad man, thus securing plenty of air and exercise. His teeth before required constant care. Now he will go sometimes for five or more years without a single cavity in his teeth. He is an unprofitable patient, because his teeth do not require any care. Dr. Black would say that they could not change for the better.

I have a young lady coming to me who first visited me four years ago. At that time she entered college with as good or better teeth than the average. I saw her the other day. She graduated in June. She had worked hard. Her teeth are a perfect wreck. A breakdown from mental strain has caused that wasting away. You may say that is due to a changed condition of the secretions of the mouth. That may be, but does not the vital condition have something to do with it. There are some things we cannot measure with the yard-stick. There are certain things we cannot demonstrate with crucible or test-tube. We say we think it is acid; we think it is bacteria; that vital force has nothing whatever to do with it. But I believe that old John Tomes was right when he said that decay was chemico-vital. Dr. Black can weigh dentine. He can tell us just how much of the various salts are in it, but that cannot determine the quality of that dentine. I understand that his experiments were made with wet dentine, not dry. He does not tell us about enamel. We all know there is a difference in the density of the enamel. I believe that enamel is living; if not, I do not know how it could change its color and its hardness, and I believe the enamel changes its hardness as well as the dentine. I have no doubt Dr. Black has made the most careful, painstaking experiments. I have the profoundest respect for a man who can go through the experiments and theories that he promulgates, but I do not believe in them. Still, I may be wrong and he may be right.

*Dr. Libby.*—I wish to say a few words in regard to what Dr. Darby has referred to,—vital force. While I have always believed in the germ theory of caries, I still maintain that vital force has much to do with it. I cannot understand how any one in the practice of dentistry for ten or twenty years can come to any other conclusion.

It undoubtedly does occur. I have often seen cases of cavities which have progressed for some time, and afterwards decay has ceased and the surface of that cavity has become brightly polished. Decay has been arrested. There has been a change in the systemic condition. The causes have been arrested by some natural operation. The caries has been arrested. How? By a change of the environment. By a change of the habits of the patient. Perhaps that patient, as Dr. Darby has just related here, has been doing a great deal of brain work. They have taken a rest from that work, from change of life, and the vital force has had a chance to repair the damage done prior to that. This, to me, is one strong argument in Dr. Darby's contention that vital forces have great influence.

*Dr. Peirce.*—Some one in Pittsburg sent me a patient who was a glass-blower. Two years ago he was elected head of the union. When he first came his teeth were just commencing to decay. But he is now in a state of nervous excitement from brain-work. I have the greatest trouble to protect his teeth. I believe it is entirely due to a systemic condition arising from irritability of the nervous system.

*Dr. Libby.*—A gentleman came to me with a loose superior bicuspid, a pronounced case of pyorrhœa alveolaris. All treatment failed and the tooth was finally extracted. A few months afterwards he came back to me with the bicuspid on the other side similarly affected. After two or three treatments the patient left me. After some months I saw him again, and was surprised to find the tooth firm and in a healthy condition. "What have you done?" I asked. "I concluded," he replied, "that you did all that you could with the other tooth, and that if I continued on with you I should have the same experience as with the first, so I bought a bottle of Hood's sarsaparilla, and this is the condition of the tooth as a result."

*Dr. Kratzer.*—Mr. President and ladies and gentlemen, as you perhaps all know, my presence here to-night is partly accidental. I was not aware, until I learned that by a lapse of memory I had missed my train and had to lay over, that I should be here to-night. This is at all times a very interesting subject, and interests us as dentists as well as patients, and I think that when we can arrive at the true theory of decay and direct our investigations or the investigations of the scientists in our profession towards the means of combating the cause by applying the antidote to the cause, we will have conferred a blessing upon humanity as well as upon our profession. I would like to mention an instance of arrest of decay without dental interference. I had occasion to extract a tooth which had become loosened by calcareous deposit, and after its extraction I found that there was a small speck resembling a filling. I examined it closely and determined in my own mind that the tooth had been filled with cement. But as the patient disclaimed ever having had a tooth filled, a closer examination compelled me to come to the conclusion that the cavity had been thoroughly filled by tartar. On drilling out the cavity this proved to be the case.

*Dr. Jefferis.*—I believe that there is some vital matter in the enamel, but so extremely small a percentage that it hardly has any weight in determining the life of the teeth in sickness or health. We do know the teeth will undergo changes physically. We know of teeth filled with gold where the fillings after ten or fifteen years will stand out from the teeth, showing that the tooth-substance has disappeared, and yet the margins will be perfectly sound around them. There has been a lessening of the diameter or the circumference of the tooth itself beneath the surface. But as to a vital change taking place in the teeth, I do not believe it.

I must accept the theory that acids and micro-organisms cause decay. I believe such changes are systemic. It may be due to the acids of the mouth, but these arise from general conditions of the blood manifested locally. The human anatomy is wonderfully complex. The saliva takes its part, and the teeth are constantly bathed in it. There are certain diseases, as of the kidneys, for instance, where the uric acid that should be eliminated is confined to the blood. We cannot go over all that ground and prove our points, but we can have our theories and believe them.

And in regard to this question of arrestation of decay without the interference of the dentist, it can be and has been done. I have seen several such cases as our friend Dr. Kratzer has mentioned, but they are not frequent. The cavity is perfectly filled and decay entirely arrested by the deposit of calcareous substance. But I hold that the improvement is due to systemic changes and not to local changes in the mouth. Nor do I believe that the changing of the tooth-structure had anything to do with the restoration.

*Dr. Roberts.*—It seems to me clinical observation and clinical experience would warrant any one in accepting the conclusion that changes take place in the teeth. They become more dense and the enamel changes color. I cannot see how Dr. Black can arrive at the conclusion that a tooth once bad is bad always. If he has had the clinical experience of a man that has been in practice any length of time he would have drawn different conclusions from his scientific observations.

*Dr. Head.*—Systemic treatment has not been dwelt upon as much as it deserves. The regulation of the teeth and preservation of the interdental spaces are also important factors. I had a case in my practice of a little girl, nine or ten years old, where the most alarming state of the teeth was present. It seemed hopeless that she should escape a severe course of tooth-regulation. She was frail and anæmic, and I concluded to prepare for the inevitable by building up the system. I gave her beef extract, syrup of hypophosphites, insisted that the child should take plenty of exercise out of doors, and, above all, I directed to be rubbed into the child, morning and evening, large quantities of cod-liver oil. To my surprise in three to six months the teeth seemed to move like magic, the jaws spread out, and in the course of a year and a half all danger of tooth-regulation passed away. Of course, nature might have come to the rescue without the treatment, but it seems to me that the treatment in this case was partly responsible for the change.

*Dr. Bonwill.*—I would propose to the dentists here, or in this city, or in the wide world, who are talking on the subject and who wish to be understood as knowing how to preserve teeth from dental caries, that they should do as Dr. Black states is the method to adopt to reach something definite and upon which we can depend in the future.

*Dr. Gilbert.*—If Professor Black is right, I cannot see how it is that for the past eighteen years I have been deceiving myself in reference to the changes in teeth. I do not see how any man with any practice at all can conceive such an idea, because teeth certainly do change, or else my observations have been very wrong.

And the other statement that it makes no difference what filling you use. Why, I think it makes all the difference what kind of filling is used in the teeth. Certain kinds of teeth are better preserved by combinations of filling than where a single kind of filling is adopted. It is surprising to me that Dr. Black will make such a statement.

*Dr. Peirce.*—I would like just to say one word. This card is a representation of sections of the enamel. The lower left hand corner will show very definitely the claims that are made. It is, as Dr. Gysi says, a transverse section of the fibres, and it gives one a very clear and definite idea of the intervening spaces, showing that certainly if you magnify the enamel fibres you do get a definite idea of the material between these fibres, and showing that the material between the fibres is organic matter.

Adjourned.

JOSEPH HEAD,
*Editor Odontological Society of Pennsylvania.*

